# The impact of hysterectomy on oncological outcomes in postmenopausal patients with borderline ovarian tumors: A multicenter retrospective study

**DOI:** 10.3389/fonc.2022.1009341

**Published:** 2022-10-27

**Authors:** Diego Raimondo, Antonio Raffone, Giovanni Scambia, Manuela Maletta, Jacopo Lenzi, Stefano Restaino, Floriana Mascilini, Rita Trozzi, Jessica Mauro, Antonio Travaglino, Lorenza Driul, Paolo Casadio, Antonio Mollo, Anna Fagotti, Giuseppe Vizzielli, Renato Seracchioli

**Affiliations:** ^1^ Division of Gynaecology and Human Reproduction Physiopathology, Istituto di ricovero e cura a carattere scientifico (IRCCS) Azienda Ospedaliero-Universitaria di Bologna, Bologna, Italy; ^2^ Department of Medical and Surgical Sciences, Department of Medical and Surgical Sciences (DIMEC), University of Bologna, Bologna, Italy; ^3^ Gynecology and Obstetrics Unit, Department of Neuroscience, Reproductive Sciences and Dentistry, School of Medicine, University of Naples Federico II, Naples, Italy; ^4^ Dipartimento di Scienze della Vita e Sanità Pubblica, Università Cattolica del Sacro Cuore, Rome, Italy; ^5^ Division of Gynecologic Oncology, Fondazione Policlinico Universitario A. Gemelli IRCCS, Rome, Italy; ^6^ Department of Biomedical and Neuromotor Sciences, University of Bologna, Bologna, Italy; ^7^ Clinic of Obstetrics and Gynecology, “Santa Maria della Misericordia” University Hospital – Azienda Sanitaria Universitaria Friuli Centrale, Udine, Italy; ^8^ University of Udine – Department of Medical Area (DAME), Udine, Italy; ^9^ Pathology Unit, Department of Advanced Biomedical Sciences, School of Medicine, University of Naples Federico II, Naples, Italy; ^10^ Gynecology and Obstetrics Unit, Department of Medicine, Surgery and Dentistry “Schola Medica Salernitana”, University of Salerno, Baronissi, Italy

**Keywords:** cancer, uterus, tumours, carcinoma, salpingo-oophorectomy, death, recurrence

## Abstract

Data about the oncological outcomes in women with borderline ovarian tumor (BOT) undergoing uterine-sparing surgery without ovarian preservation are poor. We aimed to assess the oncological outcomes in women with BOT undergoing uterine-sparing surgery without ovarian preservation. A multi-center observational retrospective cohort study was performed including all consecutive postmenopausal patients who underwent surgical treatment for BOT at three tertiary level referral centers for gynecologic oncology from January 2005 to December 2016. Patients were divided into two groups for comparisons: patients undergoing hysterectomy (hysterectomy group) and patients undergoing uterine-sparing surgery (no hysterectomy group). Study outcomes were disease-free survival (DFS), overall survival (OS), disease-specific survival (DSS) and surgical complications rate. Ninety-eight patients were included: 44 in the hysterectomy group and 54 in the no hysterectomy group. The 5- and 10-year DFS rates were 97.7% (95% CI: 84.9–99.7) and 92.3% (95% CI: 69.7–98.2), in the hysterectomy group, and 86.8% (95% CI: 74.3–93.5) and 86.8% (95% CI: 74.3–93.5), in the no hysterectomy group, respectively, without significant differences (p=0.16). Hazard ratio for DFS was 0.26 (95% CI: 0.06–1.68) for the hysterectomy group. The 5- and 10-year OS rates were 100.0% (95% CI: -) and 100.0% (95% CI: -), in the hysterectomy group, and 98.2% (95% CI: 87.6–99.7) and 94.4% (95% CI: 77.7–98.7), in the no hysterectomy group, respectively, without significant differences (p=0.23). No significant difference in complication rate was reported among the groups (p=0.48). As hysterectomy appears to not impact survival outcomes of women with BOT, it might be avoided in the surgical staging.

## Introduction

Borderline ovarian tumors (BOT) account for 15% of all ovarian epithelial tumors (1) and are characterized by atypical epithelial proliferation and absence of stromal invasion (2). In contrast to patients with invasive ovarian carcinoma, BOT has a good prognosis, with a 10-year overall survival rate of 97% (3, 4).

Treatment consists of surgery, ranging from unilateral cystectomy or salpingo-oophorectomy alone to bilateral salpingo-oophorectomy, hysterectomy and omentectomy, based on histotype, tumor stage, and desire to maintain fertility (5). Several studies have investigated the impact of hysterectomy on the survival outcomes of women with BOT (1, 3, 6–15). In fact, if avoiding hysterectomy is essential for fertility-sparing treatment of BOT, it can also have an impact in menopausal women in term of decrease of complications and surgery complexity, time and costs (16). However, these studies have reported non-univocal findings, with the consequence that international guidelines differ for the indications to hysterectomy in these women (5, 17, 18). Therefore, recently, we tried to clarify the impact of hysterectomy on survival outcomes in women with BOT through a systematic review and meta-analysis (19). We found that women with BOT who underwent hysterectomy had a significantly lower risk of recurrence than those undergoing uterine-sparing surgery, while no significant difference in the risk of death due to BOT or due to any cause was reported (19). Unfortunately, we were unable to include in the quantitative analysis only patients undergoing uterine-sparing surgery without ovarian preservation. In fact, ovarian preservation has been reported to increase BOT recurrence rate (20). Thus, the impact of uterine preservation alone is still unclear to date.

In this study, we aimed to assess the oncological outcomes of women with BOT undergoing uterine- sparing surgery without ovarian preservation.

## Materials and methods

### Study protocol and selection criteria

This was a multicentric, observational, retrospective, cohort study following an *a priori* defined study protocol. The whole study was reported according to the STrengthening the Reporting of Observational Studies in Epidemiology (STROBE) statement and checklist (21).

Medical records and electronic clinical databases were searched for all consecutive postmenopausal patients who underwent surgical treatment for BOT at three tertiary level referral centers for gynecological cancer (S. Orsola Hospital, University of Bologna, Bologna, Italy; Policlinico Gemelli, Catholic University of the Sacred Hear, Rome, Italy; University Hospital “Santa Maria della Misericordia” University of Udine, Udine, Italy) from January 2005 to December 2016. Exclusion criteria were: ovarian preservation, previous hysterectomy, coexistent endometrial cancer, invasive ovarian carcinoma on the final surgical specimen, patients referred to the tertiary level referral center for exclusive follow-up, recurrence or completion surgery, and patients with less than 5 years of follow-up (22).

Patients were divided into two groups based on uterine-sparing surgery: patients undergoing hysterectomy (hysterectomy group) and patients undergoing preservation of the uterus (no hysterectomy group).

Age at diagnosis, body mass index (BMI), parity, comorbidity (according to American Society of Anesthesiologists system and ECOG performance status (23, 24), personal history of abdominal and adnexal surgery, pretreatment CA-125, CA 19-9, CEA, CA 15-3 level (U/ml) and complications were extracted from medical records and surgical reports.

Assessed tumor characteristics consisted of tumor size (the largest tumor diameter in case of bilateral lesions), histologic type [assessed on final surgical specimen by experienced gynecological pathologists according to WHO criteria (25)] and International Federation of Gynecology and Obstetrics (FIGO) stage. Staging of the disease was retrospectively performed according to the FIGO staging system for ovarian cancer established in 2014 (26). In case of incomplete surgical staging, the stage was extracted from surgical and pathologic findings, considering unexplored abdominal areas negative for peritoneal implants.

Follow-up information were obtained from medical records and electronic clinical databases. The follow-up evaluation included gynecologic examination, transvaginal ultrasound, and serum CA-125 levels every 6 months for the first 5 years and then yearly. A follow-up of at least 5 years was performed.

### Study outcomes

Primary study outcome was disease-free survival (DFS) or time to recurrence, defined as time from surgery until there was evidence of recurrent disease confirmed at histological examination.

Secondary study outcomes were:

- overall survival (OS) or time to death, defined as time from surgery until death of any cause;
- disease-specific survival (DSS) or time to death from disease, defined as time from surgery until death due to BOT;
- surgical complications rate.

### Statistical analysis

Categorical variables were summarized as counts and percentages, while numerical variables were summarized as mean ± standard deviation and median [interquartile range (IQR)]. Differences in baseline characteristics between the two study groups were assessed with the chi-squared test, Fisher’s exact test, Student’s t-test and Mann–Whitney test, where appropriate. Differences in OS, DSS and DFS were analyzed and illustrated with the Kaplan–Meier method and log-rank test, using the date of surgery as the time origin and right-censoring patients lost to follow-up at the time of the status last known. Lastly, we used a Cox proportional hazards model to risk-adjust the association between hysterectomy and survival by including in the regression model the baseline characteristics differently distributed in the two study groups with a significance level of 0.10; the prognostic power of covariates was expressed by hazard ratios (HRs) with 95% confidence intervals (CIs). The proportional-hazards assumption was confirmed after checking for nonzero slope of scaled Schoenfeld residuals on time (27). All analyses were carried out using Stata 17 (StataCorp. 2021. Stata Statistical Software: Release 17. College Station, TX: StataCorp LLC). The significance level was set at 0.05, and all tests were two-sided.

### Ethical statement

The study received approval from the Institutional Review Board of the IRCCS Azienda Ospedaliero-Universitaria di Bologna (CE-AVEC 827/2021/Oss/AOUBo) and was carried out in accordance with the Helsinki Declaration. All patients signed an informed consent for the use of their data for the study with previous anonymization.

## Results

### Study population

During the study period, 128 postmenopausal women underwent surgical treatment for BOT. Thirty patients were excluded from the study analyses since they did not meet the selection criteria. Finally, a total of 98 patients were included in our study: 44 (44.9%) patients underwent hysterectomy and 54 (55.1%) patients spared the uterus. Ninety-six (98%) patients underwent BSO while 2 (2%) patients underwent only USO since they had been undergone a previous USO for benign disease. No patient underwent adjuvant treatment.

Mean age ± SD of patients was 56.7 ± 10.6 years in the no hysterectomy group and 64.5 ± 10.7 years in the hysterectomy group (p<0.001). No statistically significant difference between the two groups was found in terms of BMI, parity, previous abdominal and/or ovarian surgery, ASA score, and ECOG performance status. CA 125 levels were significantly higher in patients undergoing hysterectomy, while no difference between the two groups were recorded for other tumor markers (**Table 1**).

**Table 1:** Demographic and clinical data of the study population.

Characteristic	All (*n* = 98)		Hysterectomy				*P*-value
			No (*n* = 54)		Yes (*n* = 44)		
**Age, y**	60.2 ± 11.3		56.7 ± 10.6		64.5 ± 10.7		0.001*
** <50**	23	(23)	20	(37)	3	(7)	
** 50–59**	26	(27)	16	(30)	10	(23)	
** 60–69**	29	(30)	13	(24)	16	(36)	
** ≥70**	20	(20)	5	(9)	15	(34)	
**Body mass index, kg/m²**							0.97
** <25**	51	(52)	28	(52)	23	(52)	
** 25.0 to <30**	30	(31)	17	(31)	13	(30)	
** ≥30**	17	(17)	9	(17)	8	(18)	
**CA 125, U/ml**	131.3 ± 530.0		35.0 ± 64.6		249.4 ± 776.2		<0.001*
**CA 19-9, U/ml**	141.3 ± 759.4		30.4 ± 81.3		277.3 ± 1121.8		0.08
**CA 15-3, U/ml**	11.8 ± 14.6		11.5 ± 16.6		12.3 ± 11.9		0.56
**CEA, U/ml**	4.9 ± 12.6		3.7 ± 10.4		6.3 ± 14.7		0.66
**Number of pregnancies**							0.31
** 0**	21	(21)	14	(26)	7	(16)	
** 1**	31	(32)	19	(35)	12	(27)	
** 2**	22	(22)	12	(22)	10	(23)	
** 3**	15	(15)	5	(9)	10	(23)	
** ≥4**	9	(9)	4	(7)	5	(11)	
**Previous abdominal surgery**							0.59
** No**	55	(56)	29	(54)	26	(59)	
** Yes**	43	(44)	25	(46)	18	(41)	
**Previous ovarian surgery**							0.38
** No**	93	(95)	50	(93)	43	(98)	
** Yes**	5	(5)	4	(7)	1	(2)	
**ASA score**							0.23
** 1**	33	(34)	22	(41)	11	(25)	
** 2**	49	(50)	25	(46)	24	(55)	
** 3**	15	(15)	6	(11)	9	(20)	
** 4**	1	(1)	1	(2)	0	(0)	
**ECOG performance status**							0.63
** 0**	87	(89)	50	(93)	37	(84)	
** 1**	6	(6)	2	(4)	4	(9)	
** 2**	3	(3)	1	(2)	2	(5)	
** 3**	2	(2)	1	(2)	1	(2)	

Values are given as mean ± standard deviation or number (%) unless otherwise noted. SD, standard deviation; ASA, American Society of Anesthesiologists; ECOG, Eastern Cooperative Oncology Group scale. We used the symbol * to highlight the statistically significant results.

The no hysterectomy group showed a significantly higher rate of early FIGO stage than the hysterectomy group (p=0.01). Moreover, among the BOT with peritoneal implants (1 BOT at FIGO stage IIB and 3 BOT at FIGO stage IIIB in the hysterectomy group, and 1 BOT at FIGO stage IIIB in the no hysterectomy group), implants were invasive only in one patient with FIGO stage IIIB disease in the hysterectomy group.

On the other hand, the median tumor size was significantly higher in the hysterectomy group (150 mm vs 60 mm, p<0.001).

Fifty-eight BOT (59%) were serous, 31 (32%) mucinous, 5 (5%) mixed, 2 (2%) endometrioid and 2 (2%) Brenner tumors; no difference between the two groups were recorded for BOT histotype. In the no hysterectomy group, 22 (41%) BOT were diagnosed on the right ovary, 25 (46%) on the left ovary and 7 (13%) were bilateral, while in the hysterectomy group 17 (39%) patients had BOT on the right ovary, 17 (39%) on the left ovary and 10 (23%) were bilateral (**Table 2**).

**Table 2:** Tumor characteristics and oncological outcomes of the study population.

Characteristic	All patients (*n* = 98)	No hysterectomy group (*n* = 54)	Hysterectomy group (*n* = 44)	*P*-value
**FIGO stage**				0.01*
**IA**	69 (70)	44 (81)	25 (57)	
**IB**	8 (8)	5 (9)	3 (7)	
**ICI**	7 (7)	0	7 (16)	
**ICIII**	9 (9)	4 (7)	5 (11)	
**IIB**	1(1)	0	1 (2)	
**IIIB**	4 (4)	1 (2)	3 (7)	
**Tumor size,** **mm, median (range)**	95 (50–170)	60 (40–100)	150 (95–220)	<0.001*
**Laterality**				0.43
**Right**	39 (40)	22 (41)	17 (39)	
**Left**	42 (43)	25 (46)	17 (39)	
**Bilateral**	17 (17)	7 (13)	10 (23)	
**Histotype**				0.14
**Serous**	58 (59)	36 (67)	22 (50)	
**Mucinous**	31 (32)	12 (22)	19 (43)	
**Mixed**	5 (5)	4 (7)	1 (2)	
**Endometroid**	2 (2)	1 (2)	1 (2)	
**Brenner**	2 (2)	1 (2)	1 (2)	
**Complications**	5 (5)	1 (2)	4 (9)	0.48
**Recurrence**	9 (9)	7 (13)	2 (5)	0.16
**Death of any cause**	2 (2)	2 (4)	0 (0)	0.23
**Death due to tumor**	0 (0)	0 (0)	0 (0)	**-**

Values are given as number (% or range) unless otherwise noted. SD, standard deviation; FIGO, Federation of Gynecology and Obstetrics. We used the symbol * to highlight the statistically significant results.

### Study outcomes

#### Survival outcomes

Median follow-up time was 6.3 years [IQR 5.1–8.8] in the hysterectomy group and 7.1 years [IQR 5.4–10.5] in the no hysterectomy group.

During the follow-up period, 2 (4.5%) patients experienced BOT recurrences (0.7 per 100 person-years, 95% CI 0.1–2.4) in the hysterectomy group and 7 (13%) patients (1.8 per 100 person-years, 95% CI 0.7–3.6) in the no hysterectomy group. In the hysterectomy group, one patient was diagnosed with serous BOT recurrence with invasive implants on vaginal stump 7 years after first surgery for stage IIIB disease, while the other patient experienced pelvic peritoneal recurrence as pseudomyxoma peritonei after 15 months since previous surgery for stage IB mucinous intestinal type disease. In the no hysterectomy group, all the recurrence reported were serous BOT: one recurrence occurred at 12 months, three at 3 years and three at 5 years after BOT first surgery. Of these patients, six had undergone previous surgical treatment for stage IB disease and one for stage IIIB disease. Except for the above-mentioned recurrence on vaginal stump, other recurrences did not show invasive peritoneal implants. No BOT recurrence implant was reported to involve the uterus. All patients experiencing recurrence underwent second surgery and had histological confirmation of relapse. No further recurrences were reported among these patients.

**Figure 1:**
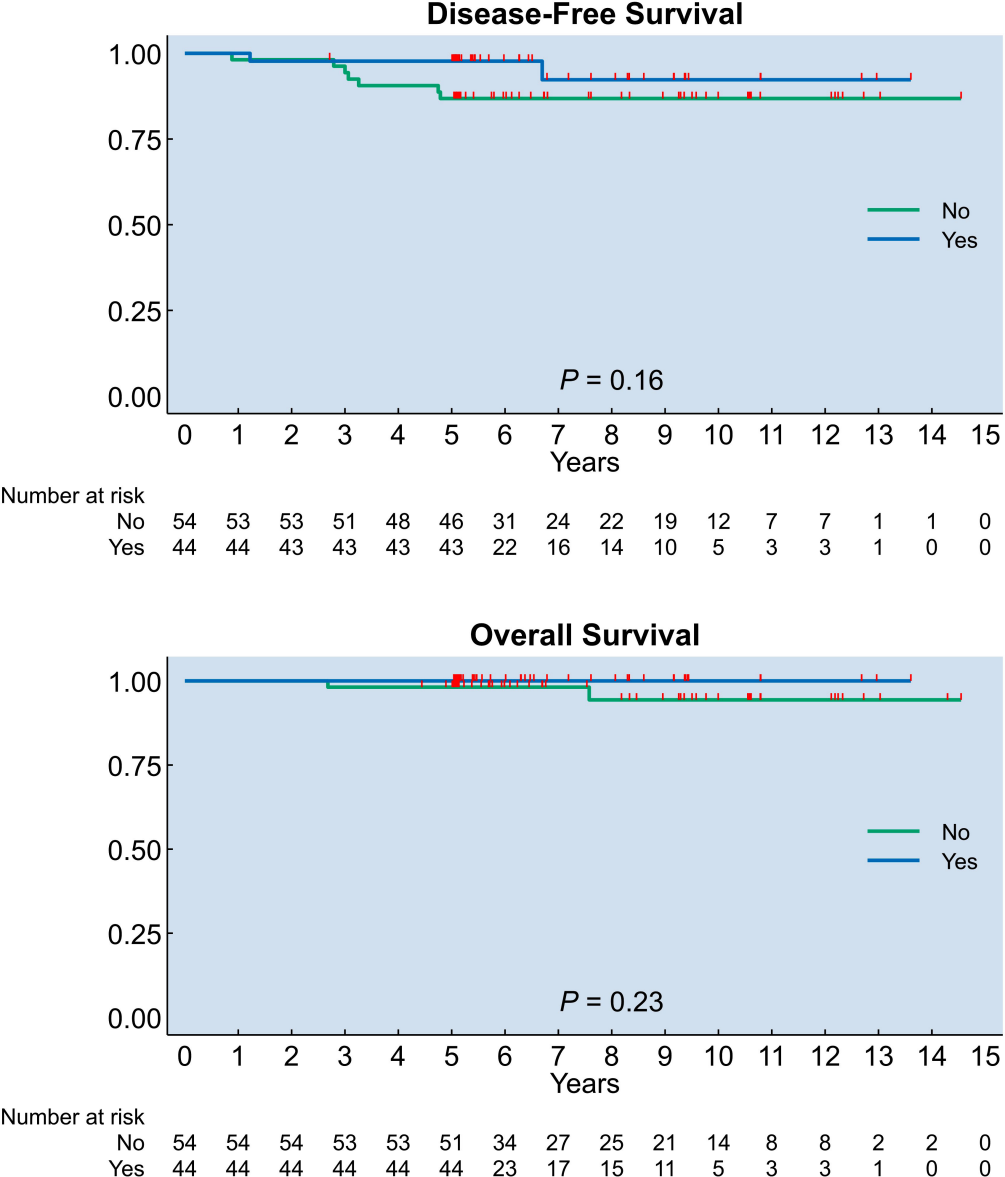
Kaplan–Meier estimates of disease-free and overall survival according to the presence of hysterectomy in the surgical treatment; censoring times are marked with red spikes. P-values for differences between groups were obtained with the log-rank test.

Kaplan-Meier curves of DFS and OS according to the presence of hysterectomy in the surgical treatment are illustrated in **Figure 1**.

The 5- and 10-year DFS rates were 97.7% (95% CI 84.9–99.7) and 92.3% (95% CI: 69.7–98.2), in the hysterectomy group, and 86.8% (95% CI 74.3–93.5) and 86.8% (95% CI: 74.3–93.5), in the no hysterectomy group, respectively (**Supplementary Table 1**). The DFS rates of the two treatment groups did not significantly differ (p=0.16; **Figure 1**). HR for DFS was 0.26 (95% CI: 0.06–1.68) for the hysterectomy group. **Table 3** shows the results of a Cox proportional hazards model used to risk-adjust the association between hysterectomy and survival for the characteristics that were differently distributed in the two study groups, i.e., age, CA 125 level, CA 19-9 level, FIGO stage, and tumor size. None of these variables was found to be a significant predictor of recurrence.

**Table 3:** Results of Cox proportional hazards model for disease-free survival.

Characteristic	Hazard ratio	95% CI	*P*-value
**Hysterectomy**			0.15
** No**	1.00		
** Yes**	0.26	0.06–1.68	
**Age, y**			0.15
** <60**	1.00		
** ≥60**	0.29	0.06–1.55	
**CA 125, U/ml**			0.32
** ≤50**	1.00		
** >50**	2.50	0.42–14.97	
**CA 19-9, U/ml**			0.92
** ≤25**	1.00		
** >25**	1.09	0.22–5.47	
**FIGO staging**			0.58
** IA**	1.00		
** IB, IC, IIB, IIIB**	1.64	0.29–9.25	
**Tumor size, mm**			0.85
** ≤100**	1.00		
** >100**	1.15	0.26–5.22	

Numerical variables were dichotomized using a median split (age and tumor size) or a split based on the 75th percentile (CA values). CI, confidence interval; FIGO, Federation of Gynecology and Obstetrics.

During the follow-up period, 2 (2%) deaths were reported: both these deaths were not due to BOT and occurred in the no hysterectomy group. The 5- and 10-year OS rates were 100.0% (95% CI: -) and 100.0% (95% CI: -), in the hysterectomy group, and 98.2% (95% CI: 87.6–99.7) and 94.4% (95% CI: 77.7–98.7), in the no hysterectomy group, respectively (**Supplementary Table 1**). The OS rates of the two treatment groups did not significantly differ (p=0.23; **Figure 1**). Since we observed only two deaths during the follow-up, multivariable regression analysis was not performed for OS.

As no death due to BOT was observed, we were unable to perform DSS analyses.

No women developed uterine malignancy during the follow-up period.

#### Surgical complications

Five patients (5%) experienced postoperative complications. In particular, 2 (4%) cases of hyperpyrexia surgically induced and 2 (4%) anemias requiring blood transfusion were reported in the hysterectomy group, while one patient (2%) undergoing uterine preservation developed fever and signs of acute abdomen. Contrast computed tomography scan showed bladder injury and a laparoscopic suture of the bladder leakage was performed. No other adverse events occurred in the following postoperative course of this patient. However, no significant difference in complication rate was reported among the groups (p=0.48).

## Discussion

### Main findings and interpretation

This study showed that women undergoing uterine-sparing surgery for BOT had not a significantly increased risk of recurrence or death of any cause compared to women undergoing hysterectomy. On the other hand, women undergoing hysterectomy had not a significant increased risk of surgical complications.

BOT are rare epithelial ovarian tumors, characterized by a good prognosis after surgical treatment, with a five-year survival rate exceeding 80% (5) and a recurrence rate ranging from 7.8% (4) to 34% (28). Within this range, several factors, such as FIGO stage, invasive and noninvasive extraovarian implants, postoperative macroscopic residual disease and conservative surgery, can affect the risk for BOT recurrence (20, 29). Regarding conservative surgical treatments, while several studies have reported the spared ovary as a risk factor for BOT recurrence (20, 30, 31), the role of the uterine preservation alone has been poorly investigated (3) up to date. In fact, if uterine-sparing surgery is necessary for preserving fertility, it can also have positively impact menopausal women management, with a decrease in complications, surgical complexity, time and costs (16). Unfortunately, recommendation for hysterectomy in surgical treatment for BOT is controversial to date. In fact, concerning surgical treatment of early-stage women with BOT not desiring pregnancy, while the National Comprehensive Cancer Network (NCCN) guidelines recommend hysterectomy for each BOT histotype (5), the Collège National des Gynécologues et Obstétriciens Français (CNGOF) recommend it exclusively for endometrioid histotype (18). On the other hand, the European Society for Medical Oncology (ESMO) - European Society of Gynaecological Oncology (ESGO) consensus conference recommendations allow for both hysterectomy and uterine-sparing surgery (17). On these bases, we tried to investigate the impact of uterine-sparing surgery on survival outcomes of women with BOT through a systematic review and meta-analysis (19). However, while we found no significant difference in the risk of death due to BOT or due to any cause (19), a significant higher risk of recurrence was highlighted in women undergoing uterine-sparing surgery. Unfortunately, such data might be affected by the presence of ovarian preservation in women undergoing uterine-sparing surgery in the primary studies, making us unable to draw definitive conclusions.

In this study we excluded women who underwent ovarian preservation and included patients only based on uterine-sparing surgery (yes/no). Surprisingly, while we found findings in accordance with the previous systematic review and meta-analysis about the risk of death of any cause and death due to BOT, data were in contrast regarding the risk of recurrence. In particular, we found that uterine-sparing surgery during BOT surgical treatment did not lead to a significantly increased risk of recurrence. This seems to mean that the previous reported increased risk of recurrence was related to ovarian rather than uterine preservation. In addition, it would be further supported by the absence of recurrence on the uterus in our cohort, in accordance with other findings in the literature reporting the rarity of uterine BOT relapse (19, 20).

Thus, as hysterectomy appears to not impact survival outcomes, it might be avoided in the surgical staging of all women with BOT, independently of pregnancy desire. In fact, although hysterectomy was found not associated to a significant increase in surgical complications rate in our study, it increases surgery complexity, time and costs (16). Moreover, the absence of significant difference in surgical complications between patients who underwent or not hysterectomy might not be confirmed in women with challenging hysterectomy (e.g. large uterus, previous surgery, previous cesarean sections, coexistent endometriosis, previous pelvic inflammatory disease), high surgical risk and/or low performance status. In this subset of patients, even a decrease in surgical complication rate might support avoiding hysterectomy.

Future larger studies are necessary to confirm and further investigate these findings.

### Strengths and limitations

To the best of our knowledge, this may be the first study to assess the impact of hysterectomy alone on survival outcomes in BOT patients. In fact, several studies have investigated the risk of recurrence among patients undergoing conservative treatment for BOT (1, 3, 6–15) including both uterine and ovarian preservation without possibility to exclusively assess uterine-sparing surgery alone (3). Moreover, another strength of our study underlies the follow-up length. In fact, we only included patients with at least 5-years follow-up, with a mean follow-up length of 6.3 years in the hysterectomy group and 7.1 years in the no hysterectomy group. Such a follow-up allows to reliably assess BOT recurrence as since up to 31.8% of BOT relapses occur after more than 5 years (22).

However, our study may be limited by the retrospective design and the relatively small sample size. However, due to the low prevalence of BOT [i.e. 15% of all ovarian epithelial tumors (1)], the retrospective design appears the most suitable study design. Moreover, about the small sample size, it is difficult to obtain a larger study population in future studies because of the study objective (i.e. to assess the oncological outcomes of women with BOT undergoing uterine-sparing surgery without ovarian preservation). In fact, most of BOT uterine-sparing surgery is performed within fertility-sparing treatment including ovarian preservation. Lastly, another limitation might be the potential understaging of patients with incomplete surgical staging: such understaging might affect survival analyses considering FIGO stage.

## Conclusion

As hysterectomy seems to not impact survival outcomes of women with BOT, it might be avoided in the surgical staging, with a decrease in surgery complexity, time and costs.

Future larger studies are needed to confirm and further investigate these findings.

## Data availability statement

The original contributions presented in the study are included in the article/**Supplementary Material**. Further inquiries can be directed to the corresponding authors.

## Ethics statement

The study received approval from the Institutional Review Board of the IRCCS Azienda Ospedaliero-Universitaria di Bologna (CE-AVEC 827/2021/Oss/AOUBo) and was carried out in accordance with the Helsinki Declaration. All patients signed an informed consent for the use of their data for the study with previous anonymization. The patients/participants provided their written informed consent to participate in this study. Written informed consent was obtained from the individual(s) for the publication of any potentially identifiable images or data included in this article.

## Author contributions

DR, AR, MM, conceived the study. JL, FM, RT, AT JM, LD, SR worked on data collection, data analysis and manuscript preparation. DR, AR, MM, PC, AM, GS, AF, GV and RS worked on the design of the study; all Authors worked on the manuscript preparation and/or revision; PC, AM, GS, AF, GV and RS supervised the whole study. All Authors approved the last version of the manuscript.

## Funding

The work reported in this publication was funded by the Italian Ministry of Health, RC-2022- 2773472.

## Conflict of interest

The authors declare that the research was conducted in the absence of any commercial or financial relationships that could be construed as a potential conflict of interest.

## Publisher’s note

All claims expressed in this article are solely those of the authors and do not necessarily represent those of their affiliated organizations, or those of the publisher, the editors and the reviewers. Any product that may be evaluated in this article, or claim that may be made by its manufacturer, is not guaranteed or endorsed by the publisher.
