# Supplementary material for: The impact of hysterectomy on oncological outcomes in postmenopausal patients with borderline ovarian tumors: A multicenter retrospective study
Source: Front Oncol. 2022 Oct 27;12:1009341. doi: 10.3389/fonc.2022.1009341 (PMC9647053; doi:10.3389/fonc.2022.1009341)
Supplement: Supplementary file 1 [file Table_1.docx]

**Supplementary Table 1.** Kaplan–Meier estimates of disease-free survival and overall survival in the first 10 years of follow-up according to hysterectomy.

|  | Hysterectomy | | | |
| --- | --- | --- | --- | --- |
| Years of follow-up | **No (*n* = 54)** | | **Yes (*n* = 44)** | |
|  | **Est.** | **95% CI** | **Est.** | **95% CI** |
| Disease-free survival |  |  |  |  |
| 1 | 98.2 | 87.6–99.7 | 100.0 | - |
| 2 | 98.2 | 87.6–99.7 | 97.7 | 84.9–99.7 |
| 3 | 96.3 | 85.9–99.1 | 97.7 | 84.9–99.7 |
| 4 | 90.6 | 78.9–96.0 | 97.7 | 84.9–99.7 |
| 5 | 86.8 | 74.3–93.5 | 97.7 | 84.9–99.7 |
| 6 | 86.8 | 74.3–93.5 | 97.7 | 84.9–99.7 |
| 7 | 86.8 | 74.3–93.5 | 92.3 | 69.7–98.2 |
| 8 | 86.8 | 74.3–93.5 | 92.3 | 69.7–98.2 |
| 9 | 86.8 | 74.3–93.5 | 92.3 | 69.7–98.2 |
| 10 | 86.8 | 74.3–93.5 | 92.3 | 69.7–98.2 |
| Overall survival |  |  |  |  |
| 1 | 100.0 | - | 100.0 | - |
| 2 | 100.0 | - | 100.0 | - |
| 3 | 98.2 | 87.6–99.7 | 100.0 | - |
| 4 | 98.2 | 87.6–99.7 | 100.0 | - |
| 5 | 98.2 | 87.6–99.7 | 100.0 | - |
| 6 | 98.2 | 87.6–99.7 | 100.0 | - |
| 7 | 98.2 | 87.6–99.7 | 100.0 | - |
| 8 | 94.4 | 77.7–98.7 | 100.0 | - |
| 9 | 94.4 | 77.7–98.7 | 100.0 | - |
| 10 | 94.4 | 77.7–98.7 | 100.0 | - |

**CI=** confidence interval.
